# The whole genome assembly and evolution analyze of carmine radish (*Raphanus sativus L.*) Mitochondrion

**DOI:** 10.1080/23802359.2020.1772136

**Published:** 2020-06-01

**Authors:** Hua Peng, Jian Gao

**Affiliations:** a College of tourism and cultural industry, Sichuan Tourism College, Chengdu, China; b Department of Life Sciences and Technology, Yangtze Normal University, Fuling, China; c Centre for Green Development and Collaborative Innovation in Wuling Mountain Region, Yangtze Normal University, Fuling, China

**Keywords:** Carmine radish, Mitochondrion genome, phylogenetic analysis

## Abstract

Carmine radish, which contained a high natural red pigment (red radish pigment), was peculiar produced in Fuling, Chongqing City. Here, the complete nucleotide sequences of the mitochondrial (mt) genome of carmine radish (*Raphanus sativus L.*) have been determined with a circular sequence with the lengths of 258,965 bp, comprised of 40 protein-coding genes, 23 tRNA genes, and three ribosomal RNA genes. To demonstrate the evolution of organelles genomes in plants, other plant mitochondrial genomes’ evolution were also selected for analyze. The results showed that carmine radish *(Raphanus sativus L.)* is related to MS_Gensuke *(Raphanus sativus L.)* and Black radish *(Raphanus sativus L.)*, as well as related to Brassica nigra and Brassica carinata, comparing with other Brassicaceae species. This study will provide important genetic tools for other Brassicaceae species research and improve yields of economically important plants.

Radish (*Raphanus sativus* L.), is an annual member of the Brassicaceae family. However, the carmine radish cultivar ‘Hongxin’ contained highly natural red pigment was scarcely reported. In the previous studies, some discordant results regarding the phylogenetic relationships between conserved relationships of radishes with different Brassica species were demonstrated through the phylogenetic analyzed. Warwick and Black showed that radish belongs to the B. rapa/Brassica oleracea lineage of subtribe Brassicinae (Warwick and Black 1991) using restriction site polymorphism obtained from analysis of several cp genomes. Contrast, a more conserved relationship with B. nigra than B. Rapa/B. oleracea were presented using a nuclear DNA marker (Yang et al. 1999).

The carmine radish (*Raphanus sativus L*.) sample (specimen number: 2018110001) used in this study was harvested from the Wuling Mountains of Chongqing (Geographic coordinate: 29_ 33 187 N, 107_ 35 3927 E), and then was deposited in plant herbarium of Department of Life Sciences and Technology, Yangtze Normal University, Fuling (Chongqing, China). The genomic DNA was extracted from etiolated seedlings according to the manufacturer’s protocol (Sangon Biotech Co., China) and the original data was constructed at the Breeding Company (Shanxi, China) on a third-generation sequencing platform. Subsequently, trimmomatic version 0.35 was used for raw sequence reads (Bolger et al. 2014). After that, two major programs comprised of MITObim (Bernt et al. 2013) and MIRA (Chevreux et al. 1999) were utilized to map the filtered reads to a reference genome (NC_018551.1) (https://www.ncbi.nlm.nih.gov/nuccore/NC_018551.1), and de novo assembly of the mitochondria genome was conducted through MITObim software (Hahn et al. 2013) and annotated with GeSeq (Tillich et al. 2017). Thereafter, the tRNA genes were confirmed through the tRNAscan-SE server (http://lowelab.ucsc.edu/tRNAscan-SE/) (Schattner et al. 2005), and the physical circular map of the mitochondria genome were drawn using the OGDRAW program (Lohse et al. 2007) with minor manual corrections. Finally, the complete mt-genome sequence was submitted to GenBank with the accession number of MT106662.1.

The results showed that the circular mitochondrial genome (258,965 bp) was assembled for carmine radish, and the overall base comprised of 26.5% A, 26.3% T, 21.7% C, and 21.6% G in descending order, and the content of A + T and G + C were found as 52.8% and 43.3% respectively. Subsequently, the structure and organization of the chloroplast genomes are identified through web-based tool Public MITOFY analysis, Of those, 66 genes comprised of 3 ribosomal RNA (rRNA) genes (5S rRNA, 26S rRNA, and rrnS), 23 transfer RNA (tRNA) genes, and 40 protein-coding genes (PCGs) were identified, as well as the positions of these genes in the carmine radish mitochondrial genome.

In addition, a phylogenetic tree obtained from the ct genome sequences of 21 species, indicated that Raphanus sativus (*carmine-radish*) is most closely related to *Raphanus sativus (MS-Gensuke) and black radish, as well as* Brassica nigra and Brassica *carinata* (especially Brassica nigra), and the *Capsella_rubella* ct genome was selected as an outgroup (Figure 1). We propose that conflicts regarding phylogenetic relationships can be resolved by using complete ct genome information, especially for ct coding genes, which could be useful for phylogenetic analyses in many closely related species and populations.

**Figure 1.**
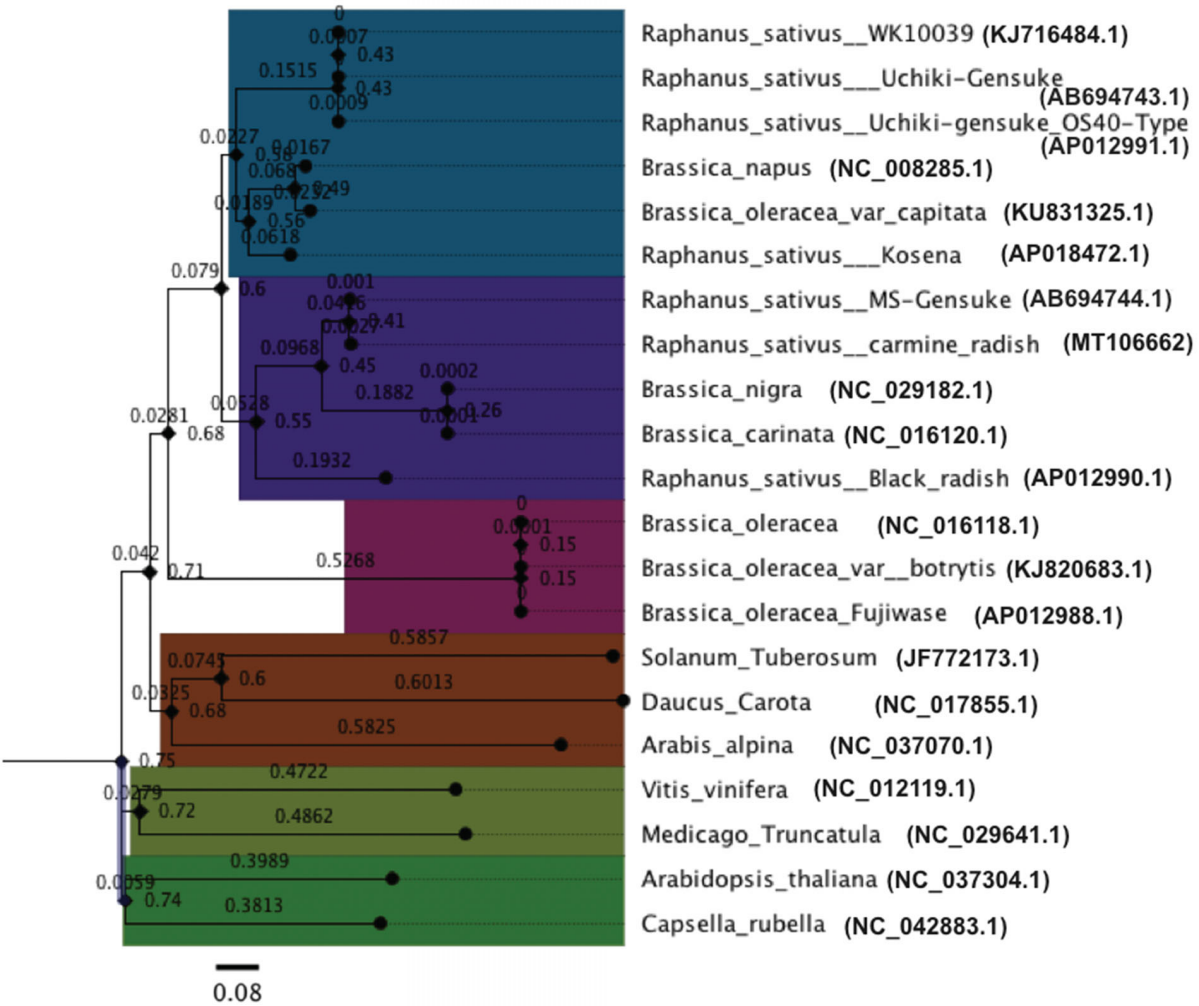
The Neighbor-Joining phylogenetic tree of 21 plant mt genomes based on conserved mitochondrial genes. Bootstrap values are listed for each node.
